# Essential thrombocytosis with recurrent spontaneous abortion in the mid trimester: A case report

**DOI:** 10.1097/MD.0000000000016203

**Published:** 2019-06-28

**Authors:** Yang Yu, Xinyue Zhang, Qingyang Shi, Meiyan Wang, Jili Jing, Yanhong Liu

**Affiliations:** Center for Reproductive Medicine, Center for Prenatal Diagnosis, First Hospital, Jilin University, Changchun, China.

**Keywords:** arrested embryonic development, essential thrombocytosis, mid trimester, recurrent spontaneous abortion

## Abstract

**Rationale:**

Essential thrombocytosis (ET) is a myeloproliferative neoplasm characterized by clonal proliferation of the megakaryocytic lineage within the bone marrow and phenotypically by an elevated platelet count in peripheral blood. Common vascular complications include thrombosis, microvascular disturbances, and hemorrhage. ET with recurrent spontaneous abortion as the primary symptom is rare.

**Patient concerns:**

A 30-year-old pregnant woman (gestational age: 8 weeks) with a history of recurrent spontaneous abortion in the mid trimester was admitted to our hospital for further management.

**Diagnosis:**

The diagnosis of ET was made based on the platelet count, bone marrow biopsy, and molecular biology testing.

**Interventions:**

The patient was treated with interferon, heparin, and aspirin.

**Outcomes:**

The infant was delivered by cesarean section without complication at 28 weeks gestation due to placental abruption. The child remained healthy with no developmental abnormalities during follow-up for 2 years.

**Lessons:**

Recurrent spontaneous abortion in the mid trimester might be associated with ET. Thus, a detailed investigation including blood routine examination to identify an abnormal platelet count is warranted for pregnant patients with such a history in order to facilitate timely treatment.

## 1 Introduction

Essential thrombocytosis (ET) is a relatively rare hematological disease. It is a clonal pluripotent stem cell disease and a type of myeloproliferative disease. ET is a myeloproliferative neoplasm (MPN) characterized by clonal proliferation of the megakaryocytic lineage within the bone marrow and, phenotypically, by an elevated platelet count in peripheral blood.^[1]^ The main clinical features are microcirculation disturbances, spontaneous bleeding tendency, and/or thrombosis. The most common clinical manifestations include thromboembolism, bleeding, headache, syncope, atypical chest pain, sensory abnormality in the extremities, visual sensory abnormality, and erythromelalgia.^[2]^ Some patients may have splenomegaly. However, approximately 50% of patients remain asymptomatic.

Due to such non-specific clinical symptoms, for young female patients, ET is often not detected until pregnancy examination. Recurrent spontaneous abortion as the primary symptom, with no history of other symptoms, is rare though. The patient whose case is presented herein had no history of thrombosis and visited our department for treatment for recurrent spontaneous abortion.

## 2 Case report

Publication of this case was approved by the Ethics Committee of the First Hospital of Jilin University. Informed written consent was obtained from the patient for publication of this case report and accompanying images.

A 30-year-old pregnant woman (gestational age: 8 weeks) with a history of recurrent spontaneous abortion presented to our hospital for management of her current pregnancy based on concerns about her previous pregnancy losses. The patient was in good health and had no history of vascular headache, dizziness, or blurred vision. She also had no symptoms of microcirculatory disturbances (e.g., acroparesthesia or acrocyanosis), fatigue, abdominal discomfort, pruritus, night sweats, bone pain, or weight loss. She had no major risk factors for cardiovascular disease and no history of vascular embolism. Her family history was unremarkable.

The patient's obstetrical status was G3P0A2. She had a history of medical abortion for intrauterine fetal death at 12 weeks gestation in 2015 and induced labor for intrauterine fetal death at approximately 16 weeks gestation in 2016.

The findings on a general physical examination were unremarkable. No hemorrhagic spots were observed on the skin or mucous membranes. She had no petechiae, and her liver and spleen were not palpable. Color Doppler ultrasound revealed no abnormality in the liver, gallbladder, pancreas, spleen, or kidneys. The spleen thickness was 30 mm at 0 mm below the coastal margin.

Both the patient and her husband underwent examinations to determine the cause of the recurrent spontaneous abortions. Both showed a normal chromosomal karyotype. The husband's seminal fluid examination showed no abnormality. The patient tested negative for anti-cardiolipin antibody and anti-β2 glycoprotein I antibody. Here serum levels of homocysteine, protein C, and protein S were normal. Her blood coagulation profile was also normal. However, her platelet count in peripheral blood was 842 × 10^9^/L, and on repeated examination, her platelet count was 915 × 10^9^/L.

Bone marrow biopsy with subsequent hematoxylin and eosin staining showed a hematopoietic area of 70% (31–49%), fat area of 10% (20–36%), and trabecular bone area of 20% (21–31%). In addition, the megakaryocyte count was 12/mm^2^ (7–15/mm^2^). Actively proliferating bone marrow nucleated cells were observed, and the granulocytic/erythroid (G/E) ratio was normal. The megakaryocyte distribution was normal, and the cells were large and multilobed. Pathological cells were seen on Periodic acid-Schiff (PAS) staining, and cells stained positively on reticular fiber staining (MF) were also observed. No abnormal change in the G/E ratio occurred on impression.

**Figure 1:**
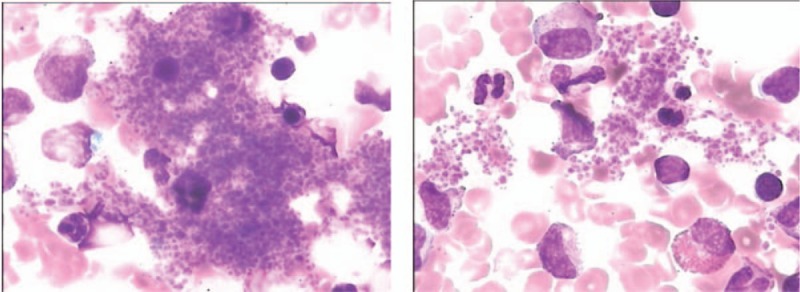
Analysis of bone marrow aspirate: actively proliferating nucleated cells; 80 megakaryocytes, including 29 classified as granular megakaryocytes and 21 as thromocytogenic megakaryocytes.

In a sample of bone marrow obtained by aspiration, actively proliferating nucleated cells were observed (Fig. 1). A granulocyte series analysis showed that 69.5% of these cells were actively proliferating, and the granulocyte ratios were normal in all stages. An erythrocyte series showed that 19% of these cells were actively proliferating. Nucleated red blood cells and mature red blood cells showed no obvious abnormalities. The lymphocyte ratio was decreased, but the morphology of the lymphocytes was normal. Examination of whole film showed 80 megakaryocytes, 50 of which were classified as granular megakaryocytes (n = 29) or thromocytogenic megakaryocytes (n = 21). Platelets were seen in large piles (Fig. 1). No abnormal cells were found in the bone marrow hematopoietic area. Iron staining was positive for external iron, and the internal iron percentage was 24%. Blood film classification showed an increase in each ratio of lobulated cells, a normal morphology among mature erythrocytes, and clustering of platelets. On impression, the G/E ratio and morphology were normal. Again, platelets were often seen in large piles.

Chromosomal examination of the bone marrow aspiration sample revealed 46, XX.^[3]^

For molecular biology analysis, genomic DNA was extracted from the bone marrow aspirate. The polymerase chain reaction (PCR) method was used to amplify target genes, and gene mutations were identified by agarose gel electrophoresis combined with gene sequencing. The patient was found to be positive for the *JAK2-V617F* mutation nd negative for the *MPL-W515L/K* and *CALR* mutations.

For qualitative analysis of BCR–ABL gene fusion, total RNA was extracted from nucleated cells of the bone marrow aspirate and reverse transcribed as cDNA. The nested PCR method was used to detect expression of the target genes and a reference gene. The results showed that the patient was negative for the BCR–ABL fusion gene. Based on these collective findings, a hematological diagnosis of ET was made, and the patient was treated with interferon, heparin, and aspirin according to standard dosing practices. The infant was delivered without complication via cesarean section at 28 weeks gestation due to placental abruption. Over 2 years of follow-up, her child remained healthy with no developmental abnormalities.

## 3 Discussion

Currently, there is no direct method for definitively diagnosing ET. According to the World Health Organization (WHO) criteria for diagnosis (2016),^[4]^ ET can be diagnosed in individuals who meet 4 major criteria or the first 3 major criteria plus a minor criterion. The major criteria include: platelet count ≥450 × 10^9^/L; bone marrow biopsy showing a high percentage of proliferating megakaryocytes with a large cell body, an increased number of large, mature megakaryocytes with hyperlobulated nuclei, no significant increase or left-shift in neutrophil granulopoiesis or erythropoiesis, and a very minor (grade 1) increase in reticulin fibers; not meeting the WHO diagnostic criteria for BCR–ABL+, chronic myelogenous leukemia (CML), polycythemia vera (PV), primary myelofibrosis (PMF), myelodysplastic syndrome, or other myeloid neoplasms; and presence of *JAK2*, *CALR*, or *MPL* gene mutations. The minor criteria include: the presence of a clonal marker or absence of evidence of reactive thrombocytosis.

ET is characterized by microcirculation disturbances, with persistently high platelet counts that cause platelet aggregation, increased blood viscosity, and tissue hypoxia. Activation of platelets leads to the production of thromboxane, which in turn induces platelet aggregation and triggers release responses, leading to microvascular thrombosis. The mechanism of thrombosis in ET has been shown to involve: procoagulant cyclic factors, such as thromboxane produced by activated platelets, which cause strong aggregation and trigger a platelet reaction, leading to microvascular embolism and thrombosis; platelets, with a histological study of erythromelalgia showing increased von Willebrand factor expression and a small amount of fibrin platelet-based arterial micro-thrombus; platelet receptor expression as well as platelet activation and platelet hemostasis reaction, which are related to the quantity and quality of the platelet surface receptor; interaction of platelets with leukocytes and endothelial cells; and hematocrit.^[5]^

Spontaneous abortion during the first trimester is the most common pregnancy-related complication in patients with ET. ET in pregnant women was reported to increase the risk of abortion by 3-fold, and many pregnant ET patients are asymptomatic.^[6]^ Published reviews have reported live birth rates of 50% to 70% and spontaneous abortion rates of 25% to 50% in pregnancy with ET.^[7,8]^ Placental micro-infarctions due to the increased platelet number and placental damage from autoantibodies are among the underlying pathological basis of first trimester abortion in ET patients.^[6]^ The detrimental effects of ET on pregnant women and fetuses are mostly microcirculation disturbances, thromboembolic disease, and bleeding tendencies in rare cases. The clinical manifestations of ET in these patients include not only spontaneous abortion but also premature delivery and fetal growth retardation.^[9,10]^ Elliott and Tefferi^[11]^ summarized the outcomes of 106 pregnancies in 57 patients with ET. Overall, 57 pregnancies (54%) were relatively successful, whereas 36% were aborted spontaneously in the first trimester, 8% ended with premature delivery, 5% experienced intrauterine stillbirth, and 4% had fetal growth retardation. In addition, a few cases had placental abruption. These complications could have been due to placental multiple thromboembolism. Vantroyen and Vanstraelen^[12]^ reviewed 27 reports that together provided data for 143 pregnancies among 75 women with ET, and the obstetrical outcomes included live birth (50.3%), spontaneous abortion (26%), premature delivery (5.6%), intrauterine fetal death in late pregnancy (9.6%), and fetal intrauterine growth restriction (5.1%). Leng et al^[13]^ reported the case of a patient with ET who experienced 4 recurrent spontaneous abortions all in the first trimester. Herein, we report the case of a woman who experienced 2 spontaneous abortions in the mid trimester and showed no genetic, autoimmune, or endocrinal abnormalities. During her third pregnancy, we were able to make the diagnosis of ET and begin appropriate treatment.

ET could be under-diagnosed during pregnancy or recur in patients with a known history, as there might be a gradual spontaneous decline in platelet count, particularly in the second trimester. The decline could be even to normal levels and greater than that in normal pregnancies (normal thrombocytopenia of pregnancy).^[14]^ A previous study suggested that pregnancy outcomes in ET patients may be correlated with the degree of platelet count reduction.^[15]^ The JAK2V617F mutation has been confirmed to be an adverse prognostic factor for pregnancy outcome in women with ET^[3]^ and may be associated with a higher rate of pregnancy complications and higher risk of second and third trimester abortion.^[16]^ Therefore, routine blood examination in early pregnancy and molecular biology analysis should be performed in patients with recurrent spontaneous abortion in order to avoid missed diagnosis of ET. Specifically, close monitoring of platelet count is useful for pregnancy management and improved outcomes in these patients.

## Author contributions

**Conceptualization:** Meiyan Wang.

**Data curation:** Xinyue Zhang.

**Formal analysis:** Yang Yu, Yanhong Liu.

**Investigation:** Xinyue Zhang, Qingyang Shi.

**Methodology:** Jili Jing.

**Software:** Jili Jing.

**Supervision:** Yanhong Liu.

**Writing – original draft:** Yang Yu.

**Writing – review & editing:** Yanhong Liu.
